# The Hair Growth-Promoting Effect of* Rumex japonicus* Houtt. Extract

**DOI:** 10.1155/2016/1873746

**Published:** 2016-11-16

**Authors:** Hyunkyoung Lee, Na-Hyun Kim, Hyeryeon Yang, Seong Kyeong Bae, Yunwi Heo, Indu Choudhary, Young Chul Kwon, Jae Kuk Byun, Hyeong Jun Yim, Byung Seung Noh, Jeong-Doo Heo, Euikyung Kim, Changkeun Kang

**Affiliations:** ^1^College of Veterinary Medicine, Gyeongsang National University, Jinju 52828, Republic of Korea; ^2^Institute of Animal Medicine, Gyeongsang National University, Jinju 52828, Republic of Korea; ^3^Gyeongnam Department of Environment Toxicology and Chemistry, Toxicity Screening Research Center, Korea Institute of Toxicology, Jinju 52834, Republic of Korea; ^4^Institutes of Agriculture and Life Science, Gyeongsang National University, Jinju 52828, Republic of Korea; ^5^Biobeauty Korea Co., Ltd., Kyungsung University Business Incubator, Busan 48434, Republic of Korea

## Abstract

*Rumex japonicus *Houtt. is traditionally used as a medicinal plant to treat patients suffering from skin disease in Korea. However, the beneficial effect of* Rumex japonicus *Houtt. on hair growth has not been thoroughly examined. Therefore, the present study aims to investigate the hair growth-promoting effect of* Rumex japonicus *(RJ) Houtt. root extract using human dermal papilla cells (DPCs), HaCaT cells, and C57BL/6 mice model. RJ induced antiapoptotic and proliferative effects on DPCs and HaCaT cells by increasing Bcl-2/Bax ratio and activating cellular proliferation-related proteins, ERK and Akt. RJ also increased *β*-catenin via the inhibition of GSK-3*β*. In C57BL/6 mice model, RJ promoted the anagen induction and maintained its period. Immunohistochemistry analysis demonstrated that RJ upregulated Ki-67 and *β*-catenin expressions, suggesting that the hair growth effect of RJ may be mediated through the reinforcement of hair cell proliferation. These results provided important insights for the possible mechanism of action of RJ and its potential as therapeutic agent to promote hair growth.

## 1. Introduction

Hair follicle is an organ composed with epithelial and mesenchymal tissues. It continuously cycles through a growth phase (anagen), an involution phase (catagen), and a resting phase (telogen) [1]. Hair loss is recently highlighted as an increasing disease characterized by the abnormal hair cycles and unstable hair follicle maintenance. Hormone imbalance, stress, nutritional deficiency, aging, and seborrheic dermatitis can cause hair loss [2–5]. Although hair loss is not life threatening, it causes serious problems (social interaction and psychology) for numerous people in the world. To date, Food and Drug Administration approved drugs in USA for the treatment of hair loss are only two, finasteride and minoxidil. Finasteride is originally developed as a treatment of benign prostatic hypertrophy but is found to have promoting effect on hair growth. It is a 5*α*-reductase inhibitor that prevents conversion testosterone to dihydrotestosterone (DHT), resulting in a decrease of androgen activity in the scalp [6]. Minoxidil is an antihypertensive vasodilator agent. It is also known to be effective in androgenic alopecia. However, the efficacy disappears within a few months after stopping therapy and side effects are reported to cause allergic dermatitis and mild scalp irritation. Use of two drugs is limited by low efficacy and unfavorable effect [7]. Therefore, the interest and importance to develop novel preventive and/or therapeutic materials for hair loss have increased recently.

The promotion of hair follicle growth is strongly influenced by various hormones, growth factors, and development-related proteins. Among them, Wnt/*β*-catenin pathway plays an important role in the hair growth cycle. *β*-catenin in DPC is markedly activated during anagen phase, resulting in initiation of hair follicle formation, stimulating hair growth and differentiation [8–10]. In *β*-catenin knock-out mice, the catagen and telogen phases induce premature condition and regeneration of hair follicle is the failure [11]. In the absence of Wnt, *β*-catenin is ordinarily phosphorylated in the cytoplasm by glycogen synthase GSK-3b (beta) and kinase-3b, tagged by an ubiquitin and then dissolved by the proteasome. However, Wnt protein binds to receptors of Frizzled family and *β*-catenin is accumulated in the cytoplasm and translocated into the nucleus, resulting in induction of growth signals for hair follicle progenitors and promoting differentiation of hair follicular keratinocyte [9, 12]. Therefore, GSK-3 is a key regulator in *β*-catenin pathway. Targeting the *β*-catenin pathway is an ideal clinical therapy for preventing hair loss and enhancing hair regrowth. Therefore, many researchers have reported chemicals and natural extracts that stimulate hair growth by targeting the *β*-catenin pathway [13–15].

*Rumex japonicus *Houtt., a perennial herb plant belonging to the family Polygonaceae, is widely distributed in the East Asia (China, Japan, and Korea). It has been used for treating heat phlegm, jaundice, constipation, uterine hemorrhage, and skin disease in traditional medicine [16–20].* R. japonicus *Houtt. contains a large number of anthraquinones, oxanthrones, and flavones and several reports demonstrate antibacterial, anti-inflammatory, and antioxidant effects in skin disease and inhibitory effect of atopic dermatitis [16, 18–23]. However, the hair growth-promoting effect of* R. japonicus *Houtt. has not been scientifically proven yet. In this study, we investigated for the first time the stimulating effect of dried roots of* R. japonicus* Houtt. extract (RJ) and its mechanism on hair growth. For this, we investigated the effect of RJ on cultured DPCs, HaCaT cells, and C57BL/6 mice.

## 2. Materials and Methods

### 2.1. Materials

Bovine serum albumin (BSA), Dulbecco's Modified Eagle's Medium (DMEM), Fetal Bovine Serum (FBS), penicillin, streptomycin, and trypsin were purchased from Gibco-BRL (Grand Island, NY, USA). Dimethyl sulfoxide (DMSO) and 3-(4,5-dimethylthiazol-2-yl)-2,5 diphenyltetrazolium bromide (MTT) were from Sigma-Aldrich Inc. (St. Louis, MO, USA). Antibodies for Bcl-2, Bax, phospho-Akt, phospho-ERK1/2, *β*-catenin, phospho-GSK3*β*, and *β*-actin were obtained from Cell Signaling Technology (Beverly, MA, USA). Minoxidil was purchased from Hyundai Pharmaceuticals (Seoul, Korea). All other reagents used were of the purest grade available.

### 2.2. Preparation of Dried Roots of* R. japonicus* Houtt. Extract

The dried roots of* R. japonicus *Houtt. were provided by Biobeauty Korea Co., Ltd. (Busan, Korea). The roots were washed 3 times with tap water to remove impurities and dried at room temperature and then stored at 4°C until use. Dried roots were ground into powder with a grinder and extracted 3 times with 95% ethanol at 25°C for 3 days, after which the extract was filtered through the Advantech number 3 filter paper (Osaka, Japan). The filtered liquid was evaporated using rotary vacuum evaporator (Tokyo Rikakikai Co., Ltd, Tokyo, Japan). The final step was lyophilization under vacuum to dryness.

### 2.3. Cell Culture

Human dermal papilla cells (DPCs) were obtained from Prof. Ohsang Kown in the Department of Dermatology, College of Medicine at Seoul National University. Human keratinocyte (HaCaT) cells were purchased from American Type Culture Collection (ATCC). DPC is obtained from occipital scalp tissue containing more than 100 hair follicles in twelve healthy male volunteers (20~50 ages, Korean) according to the approval of Seoul National University Hospital Institutional Review Board (IRB number H-1112-096-390). Before starting this experiment the DPC was cultured for three passages. They were maintained in DMEM supplemented with 10% FBS and 100 *μ*g/mL penicillin-streptomycin-amphotericin B solution. They were grown as monolayer cultures and kept at 37°C in a humidified atmosphere with 5% CO_2_ for growth.

### 2.4. MTT Assay

Cell viability was measured by the MTT assay as previously described [24]. Briefly, the cells were plated at a density of 4 × 10^4^ cells/well in 24-well plates and cultured overnight in growth DMEM media. After changing low serum media (1% FBS), the cells were treated with various concentrations of RJ and incubated for 1 and 3 days. MTT solution (5 mg/mL) was then added to each well and incubated for additional 3 h at 37°C. Finally, DMSO was added to solubilize the formazan salt and the amount of formazan generated was determined by measuring optical density (OD) at 540 nm using GENios® microplate spectrophotometer (PowerWave™XS, BioTek Instruments, Inc., Winooski, USA).

### 2.5. Western Blot Analysis

Cells plated on microplate dishes were incubated with various concentrations of RJ and then washed with cold PBS. The treated cells were collected by scraping with 300 *μ*L of RIPA buffer (iNtRON biotechnology, Seongnam, Korea) containing protease inhibitor cocktail (Thermo Fischer Scientific Pierce, IL, USA). The scraped cells were allowed to lyse for additional 30 min on ice with periodic vortexing. Cell debris was removed by centrifugation (13,000 ×g, at 4°C for 30 min) and the resulting supernatant was collected and determined for its protein concentration using a Bio-Rad protein assay reagent (Bio-Rad, CA, USA). The samples were separated on 10% SDS-polyacrylamide gel and then transferred to PVDF membranes (Bio-Rad, CA, USA). Western blot was probed with specific primary antibodies overnight at 4°C. After washing, the membranes were incubated with horseradish peroxidase-conjugated secondary antibody (Bethyl Laboratories Inc., Montgomery, USA) for 1 hr at room temperature. The blots were visualized by using an enhanced chemiluminescence method (ECL, Amersham Biosciences, Buckinghamshire, UK) and analyzed using ChemiDoc XRS (Bio-Rad, CA, USA). Densitometry analysis was performed with a Hewlett-Packard scanner and NIH Image software (Image J).

### 2.6. Immunofluorescence Analysis

The DPC and HaCaT cells were grown on cell culture slide (SPL Life Science, Pocheon, Korea) to 40–50% confluence. After incubation for 24 h, the cells were treated with RJ for another 24 h and then washed with PBS three times for 5 min. The cells were fixed with 4% paraformaldehyde for 10 min and blocked with 3% FBS for 1 h at room temperature (RT). Primary antibody (*β*-catenin) was added to the cells and incubated overnight at 4°C. The following morning, the slides were washed three times in PBS and incubated with goat anti-rabbit immunoglobulin conjugated with red-fluorescent dye for 1 h at RT. Subsequently, the stained cells were dropped with Vectashield mounting medium containing DAPI to counterstain cell nuclei (Vector Laboratories, CA, USA). The samples were observed with a Leica fluorescence microscope (Leica, Wetzlar, Germany).

### 2.7. Experimental Study with RJ for Hair Growth

This study was performed by the Korea Institute of Toxicology (Jinju, Korea) in accordance with the Institutional Animal Care and Use Committee (IACUC, 1504-0003). 6-week-old C57BL/6 mice were purchased from Samtako Inc. (Osan, Korea) and adapted for 1 week. The dorsal portion of 7-week-old mice was shaved with an animal clipper, at which mouse hair follicles were synchronized in the telogen stage. Forty animals in 4 randomized groups (*n* = 10) were used for studying the hair growing activity of RJ. RJ was dissolved in a vehicle mixture (methanol : PBS, 60 : 40% v/v). The animals in group 1 were topically treated with vehicle mixture, animals in groups 2 and 3 were treated with RJ 4 and 8 mg/mL on dorsal skin once a day for 25 days. Animals in group 4 (positive control group) were treated with 5% minoxidil in the same schedule with RJ treatment. Animals were kept in isolation for a certain amount of time and then housed back to separate cages. To assess changes in hair morphology, the mice were anesthetized and photographed at 0, 6, 11, 16, 21, and 26 days. The ratio of hair growth (grown area/shaved area) on the photographs was analyzed using image analysis program NIS-Elements Basic Research Software (Nikon Instruments INC., Tokyo, Japan).

### 2.8. Histological Examination of Hair Follicles

After 26 days, the mice were sacrificed and their shaved skins were collected. The dorsal skin was cut into two parts for hematoxylin and eosin (H&E) staining and immunohistochemistry (IHC) uses. The skin was fixed with 10% neutral formalin and embedded in paraffin blocks to obtain longitudinal and transverse section. 5 *μ*m tissue sections were stained with hematoxylin and eosin (H&E) to confirm cycle of hair follicle. The percentage of hair follicles in each defined cycle stage at day 26 was calculated. The other part of the tissue was frozen in liquid nitrogen for immunohistochemistry.

### 2.9. Immunohistochemistry

IHC for Ki-67 and *β*-catenin was performed using rabbit monoclonal antibodies. Immunoreactivity was visualized with an avidin-biotin peroxidase reaction (PK-4001, Vectastain ABC kit). The peroxidase reaction was developed using a 3, 3′-diaminobenzidine tetrahydrochloride (D-5905, Sigma). The sections were counterstained with hematoxylin before being mounted.

### 2.10. Hair Morphological Change Using Scanning Electron Microscope (SEM)

The samples were placed on an adhesive carbon tape attached to a metal stub and coated with platinum using a sputtering device. Photomicrographs of hair fiber were obtained by a SEM using JSM-7610F (JEOL, Tokyo, Japan).

### 2.11. Statistical Analysis

The results are expressed as mean ± standard deviation (S.D.). One-way analysis of variance (ANOVA) was used to evaluate the significance of difference between two mean values. *p* < 0.01 was considered to be statistically significant.

## 3. Results

### 3.1. RJ Promotes DPC and HaCaT Cells Proliferation

**Figure 1:**
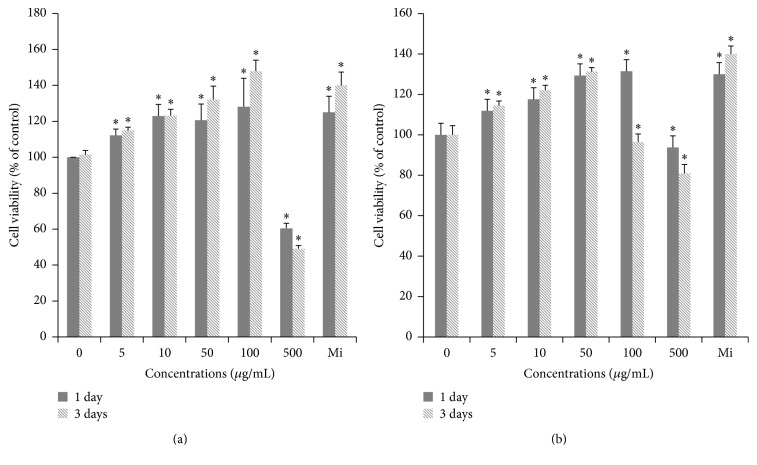
Effect of RJ on proliferation in DPC and HaCaT cells. (a) DPC and (b) HaCaT cells were treated with RJ (5, 10, 50, 100, and 500 *μ*g/mL) for 1 and 3 days. Cell viability was then determined in a MTT assay. Minoxidil (Mi, 100 *μ*M) was used as positive control. The data shown are the mean ± SD of three independent experiments. Significant difference from control group, ^*∗*^ *p* < 0.01.

DPC and HaCaT cells were used as models in this study because they are important in the growth of hair. To determine the effects of RJ on DPC and HaCaT cells, MTT assay was performed. In addition, 100 *μ*M minoxidil (Mi) was used as a positive control. RJ enhanced the proliferation of DPCs by 132.1 and 147.9% at the concentrations of 50 and 100 *μ*g/mL for 3 days compared with the control (Figure 1(a)). In DPC, treatment of RJ 100 *μ*g/mL for 3 days exhibited the greatest effectiveness, which is stronger than Mi. RJ treatment significantly also promoted the proliferation of HaCaT cells by 131.3% at 50 *μ*g/mL for 3 days compared with the control (Figure 1(b)). The results suggest that RJ might have hair growth-promoting effect via the proliferation of DPC and HaCaT cells.

### 3.2. RJ Activates Akt, ERK1/2, and Wnt/*β*-Catenin in DPC and HaCaT Cells

**Figure 2:**
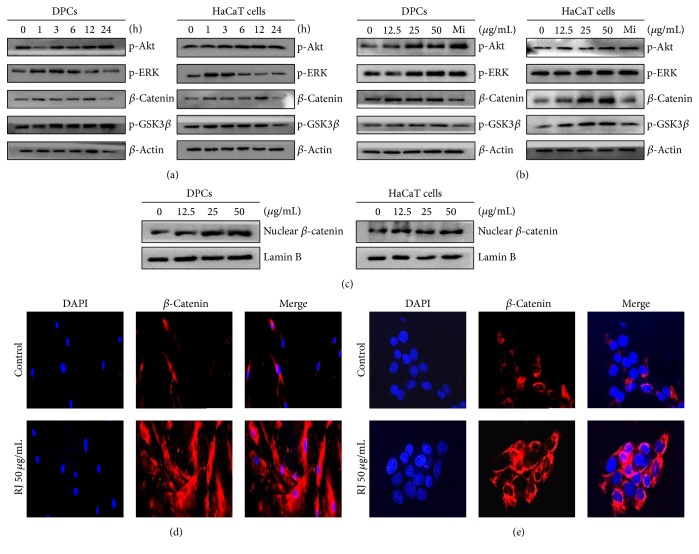
Effect of RJ on ERK1/2, Akt, p-GSK3*β*, and *β*-catenin expressions in DPC and HaCaT cells. (a) Time-dependent effects of p-ERK1/2, p-Akt, p-GSK3*β*, and *β*-catenin expressions were determined in cells treated with RJ (50 *μ*g/mL) for the indicated times. (b) Dose-dependent effects of p-ERK1/2, p-Akt, p-GSK3*β*, and *β*-catenin were detected in RJ-treated cells using western blotting. (c) To confirm translocation of *β*-catenin into nucleus, nuclear *β*-catenin expression was determined by western blotting. (d and e) The *β*-catenin expression was determined by immunofluorescence staining with anti-*β*-catenin in DPC and HaCaT cells.

Minoxidil has been well known to enhance proliferation of hair cells through MAPK kinase family (ERK1/2, JNK1/2, and p38) and Akt signaling pathways. Therefore, the mechanism of proliferative effects by RJ in DPC and HaCaT cells was determined by western blot. As shown in Figure 2(a), the expression level of p-Akt was steadily increased for up to 24 h in time-dependent manner on both DPC and HaCaT cells, while p-ERK1/2 level was elevated up to 6 h in DPC and 1 h in HaCaT cells by treatment of RJ 50 *μ*g/mL. The phosphorylation of JNK1/2 and p38 did not change in DPC and HaCaT cells (data not shown). Additionally, RJ increased the expressions of *β*-catenin and p-GSK3*β* for 12 h in both DPC and HaCaT cells. Treatment with dose-dependent manner of RJ increased the p-Akt and p-ERK1/2 as well as *β*-catenin and p-GSK3*β* in DPC and HaCaT cells for 24 h (Figure 2(b)). Nuclear *β*-catenin expression also was increased by RJ treatment in both cells (Figure 2(c)). Interestingly, the expression levels of *β*-catenin and p-GSK3*β* were greatly elevated by treatment with RJ, but not in Mi. Moreover, we confirmed significant activation of *β*-catenin in DPC and HaCaT cells treated with RJ 50 *μ*g/mL by immunofluorescence. In control cells, *β*-catenin was very weakly detected in both cells. However, its expression was increased and detected in cytoplasm by treatment of RJ (Figures 2(d) and 2(e)). Similarly with western blotting result, expression of *β*-catenin was increased by RJ in immunofluorescence data. These results suggest that RJ can increase the proliferation of DPC and HaCaT cells via ERK1/2, Akt, and *β*-catenin signaling pathways.

### 3.3. Regulation of RJ on Apoptosis-Related Proteins

**Figure 3:**
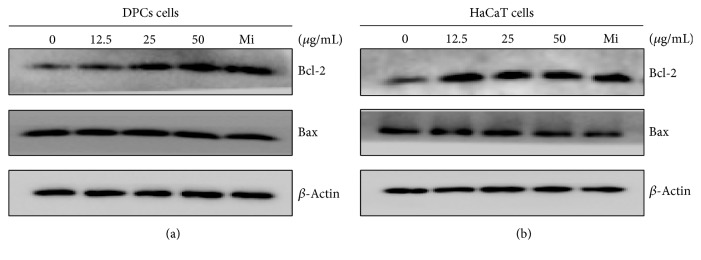
Effects of RJ on expressions of Bcl-2 and Bax proteins. (a) DPC and (b) HaCaT cells were treated with RJ (0, 12.5, 25, and 50 *μ*g/mL). The levels of Bcl-2 and Bax were analyzed by western blotting using specific monoclonal antibodies. *β*-Actin was used as a loading control. The data shown are the mean ± SD of six independent experiments. Significant difference from control group, ^*∗*^ *p* < 0.01.

Since RJ induced the growth-promoting effect of DPC and HaCaT cells, we examined the expression levels of the anti- and proapoptotic protein in the two Bcl-2 family proteins. In response to treatment of DPC with RJ, the level of antiapoptotic Bcl-2 protein dose-dependently increased by 1.2- to 2-fold and the level of proapoptotic Bax protein significantly decreased at only 50 *μ*g/mL (Figure 3(a)). Additionally, in HaCaT cells, RJ induced significant upregulation of Bcl-2 protein and downregulation of Bax protein in dose-dependent manner (Figure 3(b)). Bcl-2/Bax ratio was increased by 1.1- to 2.4-fold and 1.2- to 2.2-fold in DPC and HaCaT cells as dose increased from 12.5 to 50 *μ*g/mL (Figures 3(a) and 3(b)).

### 3.4. RJ Promotes Induction of Anagenic Phase in Telogenic C57BL/6 Mice

**Figure 4:**
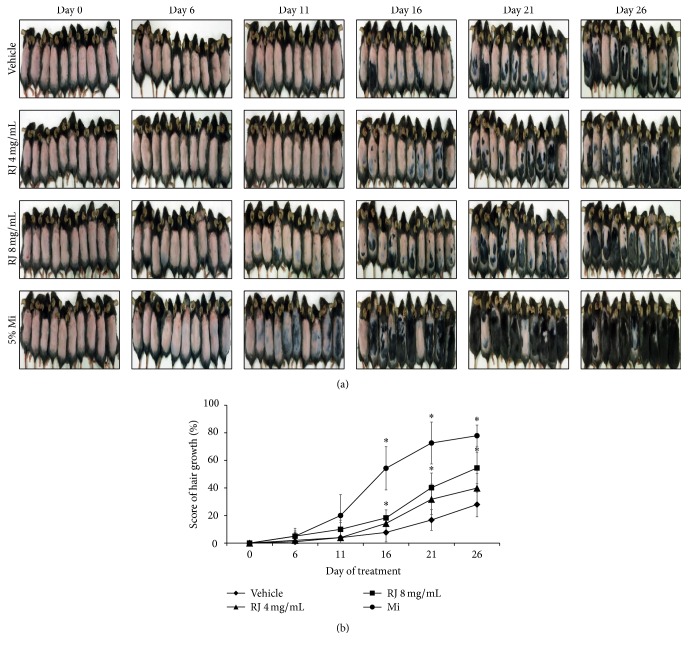
Effect of RJ on the anagen induction in C57BL/6 mice. (a) After shaving, the back skin was treated with vehicle, RJ, and 5% Mi every day for 25 days. The back skin was photographed at 0, 6, 11, 16, 21, and 26 days after shaving. (b) Morphological change in C57BL/6 mice treated with vehicle, RJ, and Mi was analyzed. Parts of the shaved and the hair growth area measured using the NIS-Elements Basic Research Software. The data shown are the mean ± SD (*n* = 10). Significant difference from vehicle group, ^*∗*^ *p* < 0.01.

The black pigmentation was taken as evidence for transition of hair follicles from telogen to anagen phase. To evaluate the hair growth-promoting activity of RJ, we topically applied daily RJ onto the shaved back of telogenic C57BL/6 mice for 25 days. 5% Mi was separately applied as a positive control. We evaluated the degree of hair growth by observing the skin color every 5 days. At 11 days, RJ-treated group showed partly black coloration and short hair in the shaved skin, while less visible black pigmentation was observed in vehicle group (Figure 4(a)). At 16 and 21 days, RJ-treated group(8 mg/mL) showed markedly hair growth over half of mice. At 26 days, in RJ-treated group (8 mg/mL), the mice back was covered with overall hair except for three mice, whereas the vehicle group showed relatively less hair growth. In the Mi-treated group, the change in the color of back skin was earlier than in the other groups and covered completely after 26 days except for two mice. The effect on hair growth of RJ treatment was assessed by measuring regrown area and shaved area. Mi-treated group showed 83.9% of hair growth score at 26 days compared with the vehicle group which kept showing 26% of hair growth score. Although less effective than Mi-treated group, RJ-treated group exerted 39.9 and 54.5% of hair growth score at 4 and 8 mg/mL at 26 days (Figure 4(b)). These results indicate that RJ promoted hair growth and telogen to anagen transition in C57BL/6 mice.

### 3.5. Effects of RJ on Development of Hair Follicle

**Figure 5:**
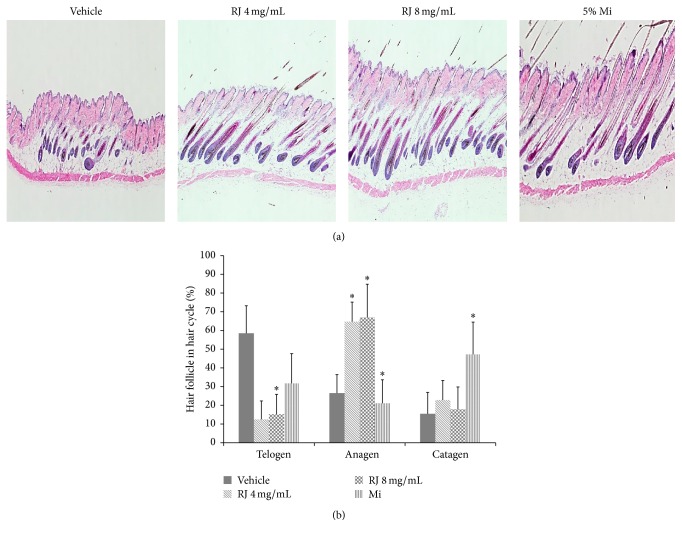
Analysis of histological change in C57BL/6 mice treated with vehicle, RJ, and minoxidil for 25 days. (a) Longitudinal sections of the dorsal skins were stained using H&E at 26 days. (b) The cycle progression of hair follicle was analyzed by counting.

An increase in the number and the size of hair follicles during anagen induction is reported [25]. To investigate whether RJ induces the anagen induction in hair follicle, H&E staining was performed. In the representative longitudinal sections, the length of hair follicle in RJ-treated group was longer and the size was larger than in the vehicle group (Figure 5(a)). Skin thickness also was increased in RJ- and Mi-treated groups compared to vehicle group. Additionally, as a result of analyzing hair cycle, RJ-treated groups showed that anagen phase of hair follicle was increased over 2-fold and telogen phase was decreased by 6-fold as compared with vehicle group. The percentage ratio of anagen to telogen phase of hair growth in RJ-treated group (8 mg/mL) was also increased from 26 : 58 to 66 : 12 in comparison with vehicle group (Figure 5(b)). These results suggest that RJ enhanced the hair growth, which appeared in the increase in the hair follicle number, size, length, and skin thickness and induction of early telogen to anagen conversion, compared to the vehicle group.

### 3.6. Involvement between the Promoting Hair Growth Effect and *β*-Catenin

**Figure 6:**
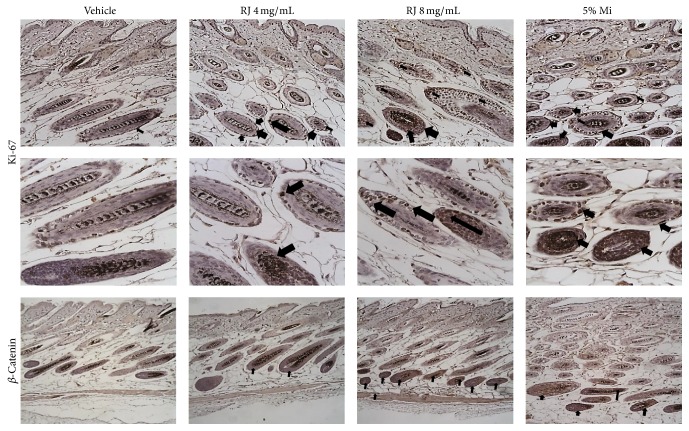
Effect of RJ on expression of hair growth-related protein. Ki-67 (arrow) and *β*-catenin expression in the nuclei and cytoplasm of proliferating follicular cells, respectively (brown color). There are hairs with melanin pigment (black color) at the center of follicles.

To confirm the proliferation and promoting effect in hair cells, we performed the IHC analysis using Ki-67, which is cellular proliferation marker [26]. Immunoreactivity to Ki-67 revealed that RJ 8 mg/mL and Mi enhanced more cellular proliferation within matrix part of hair follicles than vehicle group (Figure 6). To understand the mechanism of inducing anagen phase in RJ-treated group, the expression of *β*-catenin was confirmed using IHC analysis. *β*-catenin protein expression levels were strongly detected in RJ 8 mg/mL compared to vehicle group (Figure 6).

## 4. Discussion

Hair loss is generally not a life-threatening disorder, but it is psychological disease that has an impact on social interaction. To date, there are only two antihair loss drugs, finasteride and minoxidil. Those drugs have been used in clinical affairs, but their efficacy is transient and they cause undesirable adverse effects. Therefore, herbal plants with hair promoting activity in alternative medicine are increasingly attracting attention due to minimal or no side effect.* R. japonicus *Houtt. has been traditionally used to treat skin disease and problems in East Asia [16]. Recently, some companies produce shampoo and soap containing an extract of* R. japonicus *Houtt. and claim that they are potential antihair loss products and promote hair growth effects. However, there is no scientific evidence to explain these beneficial effects of RJ on hair growth. Hence, in this study, we investigated the hair growth-promoting effect of RJ using DPC, HaCaT cells, and C57BL/6 mice.

The DPC are major hair components and secrete a number of growth factors that can modulate the proliferation of follicular epithelium and follicle development [27]. Several studies demonstrated that the size of DPC is well correlated with the hair growth and the number of DPC increases in the early anagen stage [28, 29]. Keratinocyte plays pivotal roles in hair follicle and hair shaft. The cells at early anagen are activated and subsequently fasted proliferating cells to make hair bulb structure with hair matrix surrounding DPC before activation of hair follicle stem cell [30]. MTT results indicated that RJ significantly stimulated proliferation by 1.3-fold in both DPC and HaCaT cells compared with nontreated cells. The proliferative effects of RJ on DPC and HaCaT cells are considered a maker maintenance and elongation of anagen phase. In agreement with* in vitro* data, the effect of RJ on hair growth was proved in* in vivo* study. The black mouse C57BL/6 is used for screening hair growth agents as an animal model for hair growth due to the fact of producing their pigmentation during only anagen [31]. The shaved back skins of C57BL/6 were treated with topical application of RJ (4 and 8 mg/mL) for 25 days. RJ induced stimulation of hair growth in the telogenic C57BL/6 mice. Mice at higher dose group (RJ, 8 mg/mL), showed significant and more abundant hair growth than those in the vehicle group and lower dose group (RJ 4 mg/mL). RJ-treated groups also showed that anagen phase was increased over 2-fold and telogen phase was decreased by 6-fold, resulting in that the ratio of anagen to telogen phase was increased from 0.5 : 1 to 5.5 : 1 in RJ-treated group, 8 mg/mL. In addition, SEM analysis revealed that hair surface of RJ-treated groups is entirely clear, smooth, and intact and is tightly overlapping cuticle without brokenness compared with vehicle group (see Supplement Figure 1 in Supplementary Material available online at http://dx.doi.org/10.1155/2016/1873746). Any adverse signs (skin irradiation, drying, and edema) were not observed at the site of application of RJ for total experimental procedure. Furthermore, the histological data indicated that RJ-treated group (8 mg/mL) showed increase in the number and the size of hair follicles, skin thickness, and an earlier induction of the anagen phase, compared with vehicle group. IHC analysis showed that Ki-67 expression was upregulated in RJ 8 mg/mL group compared to that in vehicle group. Collectively, RJ promoted hair growth and anagen induction via proliferation of hair follicle-related cells and the length and size of hair follicle in* in vivo* study.

To elucidate the molecular mechanism underlying the ability of RJ to induce hair growth- promoting effect, the protein levels of MAPK kinase family and Akt, which are hair follicle cell proliferation-related molecules, were checked in the DPC and HaCaT cells. Several reports demonstrated that hair growth agents promoted the proliferation and survival of DPC through the activation of ERK1/2 and Akt [32–34]. The ERK signaling is known to be activated by mitogens in all types of mammalian cells and its key roles in cell growth have been established previously [35]. Furthermore, it was recently demonstrated that ERK activation plays an important role in the proliferation of DPC [33]. Akt also mediates critical signals for cell survival and regulates the survival of DPC as antiapoptotic molecule [32]. Western blot data showed that the effect of RJ on hair growth was associated with activation of ERK1/2 and Akt signaling. RJ significantly increased ERK1/2 expression levels in both DPC and HaCaT cells. Therefore, the activation of Akt by RJ might prolong the survival of DPC and HaCaT cells. In addition, expressions of Bcl-2 and Bax indicated that RJ prevented cell death in DPC and HaCaT cells.

Recently, among the development-related molecules in hair follicles, it is known that *β*-catenin seems to be critical in hair loss. Several studies have suggested a relationship between *β*-catenin signaling and alopecia. When androgen treated DPC in androgenic alopecia patients, *β*-catenin was significantly decreased in the cytoplasm, while GSK-3*β* was increased [36, 37]. BIO (GSK-3*β* inhibitor) enhances hair-inducing ability through stabilization of *β*-catenin and elevated the alkaline phosphatase and insulin-growth factor-1 gene expressions [38, 39]. In addition, *β*-catenin is key regulator of hair follicle growth and cycling phase [40]. During morphogenesis of hair follicle, elevated expression of *β*-catenin-dependent genes is observed. Deletion of *β*-catenin inhibits formation of hair follicle, while constitutively activated *β*-catenin produces an expansion of hair follicle fate [41–43]. *β*-catenin also induces the transition of hair follicles from the telogen phase to anagen phase. Hair cycle regeneration and anagen phase maintenance are *β*-catenin dependent [10, 11]. Therefore, it was speculated whether RJ induced the hair growth-promoting effect through *β*-catenin pathway. Western blot data showed that the expression levels of *β*-catenin were upregulated by RJ-treated in DPC and HaCaT cells, compared to those in the control or Mi-treated cells. IHC analysis results showed that the expression of *β*-catenin was increased in RJ-treated cells. Furthermore, in* in vivo* study, there was a significant increase of *β*-catenin expression in RJ-treated group (8 mg/mL) compared with vehicle group. On the other hand, it is recently reported that activation of ERK1/2 and Akt is related to *β*-catenin signaling pathway [44, 45]. Their phosphorylation increased *β*-catenin/TCF complex through inactivation of GSK3*β*, indicating that phosphorylation of ERK1/2 and/or Akt induces activation of *β*-catenin signaling pathway. Therefore, the increase of activation of ERK1/2 and Akt by treatment of RJ might help proliferation of hair cells as well as activation of *β*-catenin, resulting in induction and maintenance of anagen.

In conclusion, although RJ has less hair growth effect compared with minoxidil, it stimulated hair growth by inducing anagen phase of hair follicles and proliferation of hair cells. We further showed new insights into possible mechanism of action by which RJ may contribute to the pharmacological intervention of alopecia therapy. However, there is no doubt that the limitations of this study still need further investigations and improvements. Additional clinical trials and studies will be necessary to investigate what components in RJ contribute to its efficacy.

## Supplementary Material

To observe the morphological changes on the hair surface, the samples were fixed in 1% osmium tetroxide and then dehydrated in an ascending ethanol series (10% to 100%). After drying, the samples were coated with platinum and observed using JSM-7610F (JEOL, Tokyo, Japan).
