# Supplementary figures and images for: The Hair Growth-Promoting Effect of Rumex japonicus Houtt. Extract
Source: Evid Based Complement Alternat Med. 2016 Nov 16;2016:1873746. doi: 10.1155/2016/1873746 (PMC5128716; doi:10.1155/2016/1873746)

**Vehicle**

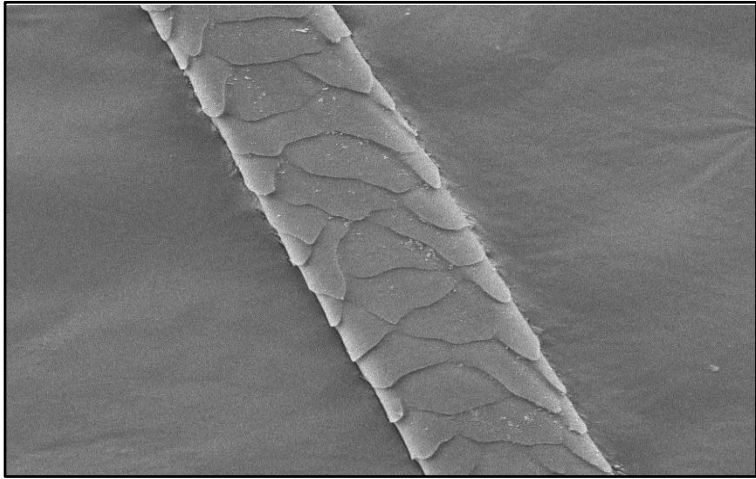

**RJ 4 mg/ml**

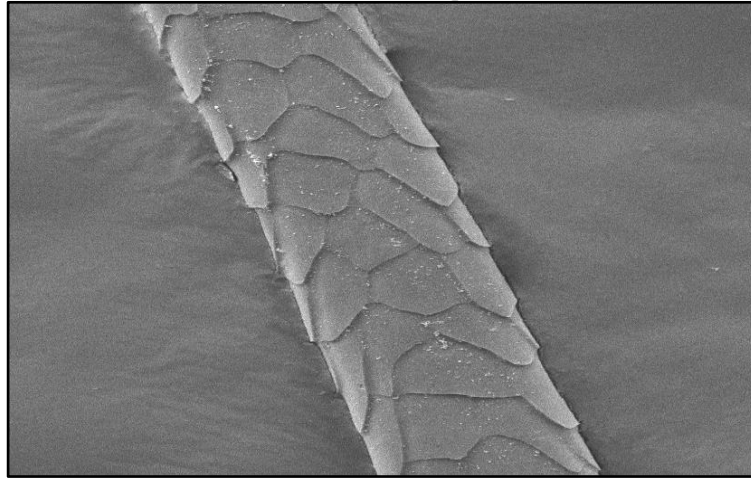

**RJ 8 mg/ml**

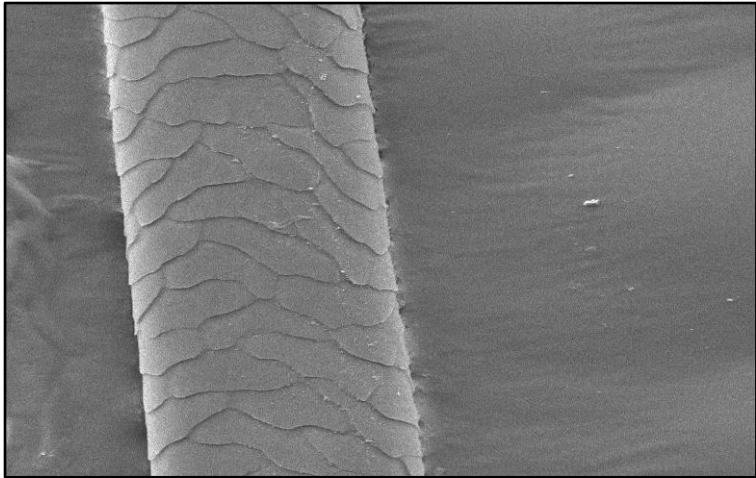

**5% Mi**

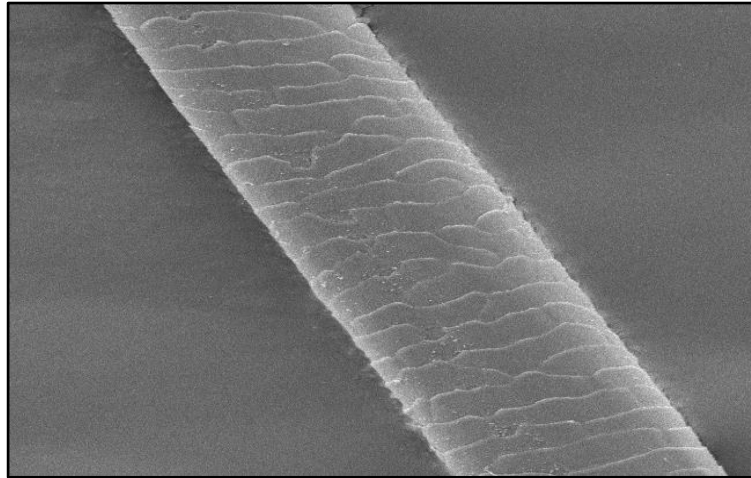

**Supplement Fig.1**

Supplement: Supplementary file 1 — To observe the morphological changes on the hair surface, the samples were fixed in 1% osmium tetroxide and then dehydrated in an ascending ethanol series (10% to 100%). After drying, the samples were coated with platinum and observed using JSM-7610F (JEOL, Tokyo, Japan). [file 1873746.f1.pdf]
